# Identification of Photoperiod-Induced LncRNAs and mRNAs in Pituitary Pars Tuberalis of Sheep

**DOI:** 10.3389/fvets.2021.644474

**Published:** 2021-08-03

**Authors:** Qing Xia, Mingxing Chu, Xiaoyun He, Qiuyue Liu, Xiaosheng Zhang, Jinlong Zhang, Xiaofei Guo, Ran Di

**Affiliations:** ^1^Key Laboratory of Animal Genetics and Breeding and Reproduction of the Ministry of Agriculture and Rural Affairs, Institute of Animal Science, Chinese Academy of Agricultural Sciences, Beijing, China; ^2^Institute of Genetics and Developmental Biology, Chinese Academy of Sciences, Beijing, China; ^3^Tianjin Institute of Animal Sciences, Tianjin, China

**Keywords:** lncRNAs, mRNAs, photoperiod, sheep, pituitary pars tuberalis

## Abstract

The pituitary pars tuberalis (PT) is the regulating center of seasonal reproduction, which can sense the melatonin signal and eventually cause downstream changes of GnRH secretion through TSHβ. Recently, lncRNAs have been identified in animal reproductive-related tissues, and they play important roles in reproductive regulation. Therefore, in this study, we expect to identify photoperiod-induced lncRNAs and genes in pituitary PT of sheep by comparison of expression profiles between short photoperiod (SP) and long photoperiod (LP). Through RNA-Seq, a total of 55,472 lncRNAs were identified in pituitary PT of Sunite ewes. The number of differentially expressed (DE) genes and lncRNAs between SP and LP increased gradually with the extension of LP (from LP7 to LP42). The notable LP-induced candidate genes included *EYA3, TSHB, SIX1, DCT, VMO1, AREG, SUV39H2*, and *EZH2*, and SP-induced genes involved *ENSOARG00000012585, CHGA, FOS, SOCS3*, and *TH*. In enriched pathways for DE genes and lncRNA target genes between SP and LP, the reproduction- and circadian-related pathways were highlighted. In addition, the interactome analysis of lncRNAs and their targets implied that *MSTRG.209166* and its *trans*-target *TSHB, MSTRG.288068* and its *cis*-target *SIX1*, and *ENSOARG00000026131* and its *cis*-target *TH* might participate in regulation of seasonal reproduction. Together, these results will help to determine important photoperiod-induced lncRNAs and genes and give us some new insights into the epigenetic regulation of seasonal reproduction in sheep.

## Introduction

The reproductive activity of some animal species inhabiting the temperate zone is limited in specific seasons in order to maximize the survival possibility of their offspring. According to the different seasons of breeding, these animals are categorized as long photoperiod (LP) breeders and short photoperiod (SP) breeders (1, 2). Among them, sheep belongs to the SP breeder (3); that is, they are mostly bred in autumn and winter. For example, Sunite sheep in China exhibit obvious seasonal reproductive behavior throughout the year, i.e., estrus from August to March of the next year and anestrus from April to July (2, 4). Seasonal reproduction is an important factor limiting the production effciency of the sheep industry; therefore, research on the molecular basis of seasonal reproduction of sheep is indispensable for possible artificial regulation of this trait in the future. However, the molecular mechanism and regulatory network of seasonal reproduction are not very clear until now.

Pituitary pars tuberalis (PT) plays an important role of transmission center in the seasonal reproduction of animals. The pineal gland first converts external photoperiod signals into biological signals (nocturnal melatonin secretion), which causes photoperiod-induced signals (*EYA3, TSHB*, etc.) alternation in PT. Thus, the thyrotrophin secreted by the anterior pituitary will increase in long days, which acts on TSH receptor-expressing cells in the adjacent mediobasal hypothalamus, leading to type III thyroid hormone deiodinase (DIO3) switch to type II thyroid hormone deiodinase (DIO2). DIO2 regulates the thyroid hormone in the hypothalamus, controlling the activity of the hypothalamus–pituitary–gonad axis to exhibit the summer phenotypes by direct (GnRH neuron) or indirect (*KISS1*/*RFRP* system) ways (5, 6). Besides, the photoperiod-induced gene expression in PT is mainly affected by photoperiod, independently of the TH status (6). However, molecular changes related to seasonal reproduction in medio-basal hypothalamus are regulated not only by photoperiod but also by TH. Therefore, PT is the best ideal tissue for seasonal reproduction analyses. So far, several important genes involved in the regulation of seasonal reproduction have been discovered in PT. For example, LP-induced genes (e.g., *EYA3* and *TSH*β) and an SP-induced gene (*CHGA*) were revealed in PT of sheep (3, 7, 8). Their expression levels had a remarkable change in different photoperiods. In recent years, long non-coding RNAs (lncRNAs) are considered as key regulators of gene expression because they play crucial roles in transcriptional and post-transcriptional regulation. In sheep, many lncRNAs have been identified in reproduction-related tissues, such as ovary, uterus, and germ cells (9–12). Moreover, lncRNAs play important roles in many aspects of sheep reproductive regulation, such as fecundity (12, 13), gonadal development (14), and sex hormone response (15). However, the functions of lncRNAs in animal PT on seasonal reproduction are unknown. Therefore, one purpose of this research is to seek the new photoperiod-induced genes and lncRNAs in PT of sheep by transcriptome sequencing. Another objective is to explore the potential relationship between genes and lncRNAs by target prediction and joint analysis of their co-expression in PT of sheep. These results will give us some new insights into the epigenetic regulation of seasonal reproduction in sheep.

## Methods

### Ethical Statement

All the animals were authorized by the Science Research Department (in charge of animal welfare issue) of the Institute of Animal Sciences, Chinese Academy of Agricultural Sciences (IAS-CAAS; Beijing, China). In addition, ethical approval of animal survival was given by the animal ethics committee of IAS-CAAS (No. IAS2018-3, April 10, 2018).

### Animal and Tissue Acquirement

**Figure 1:**
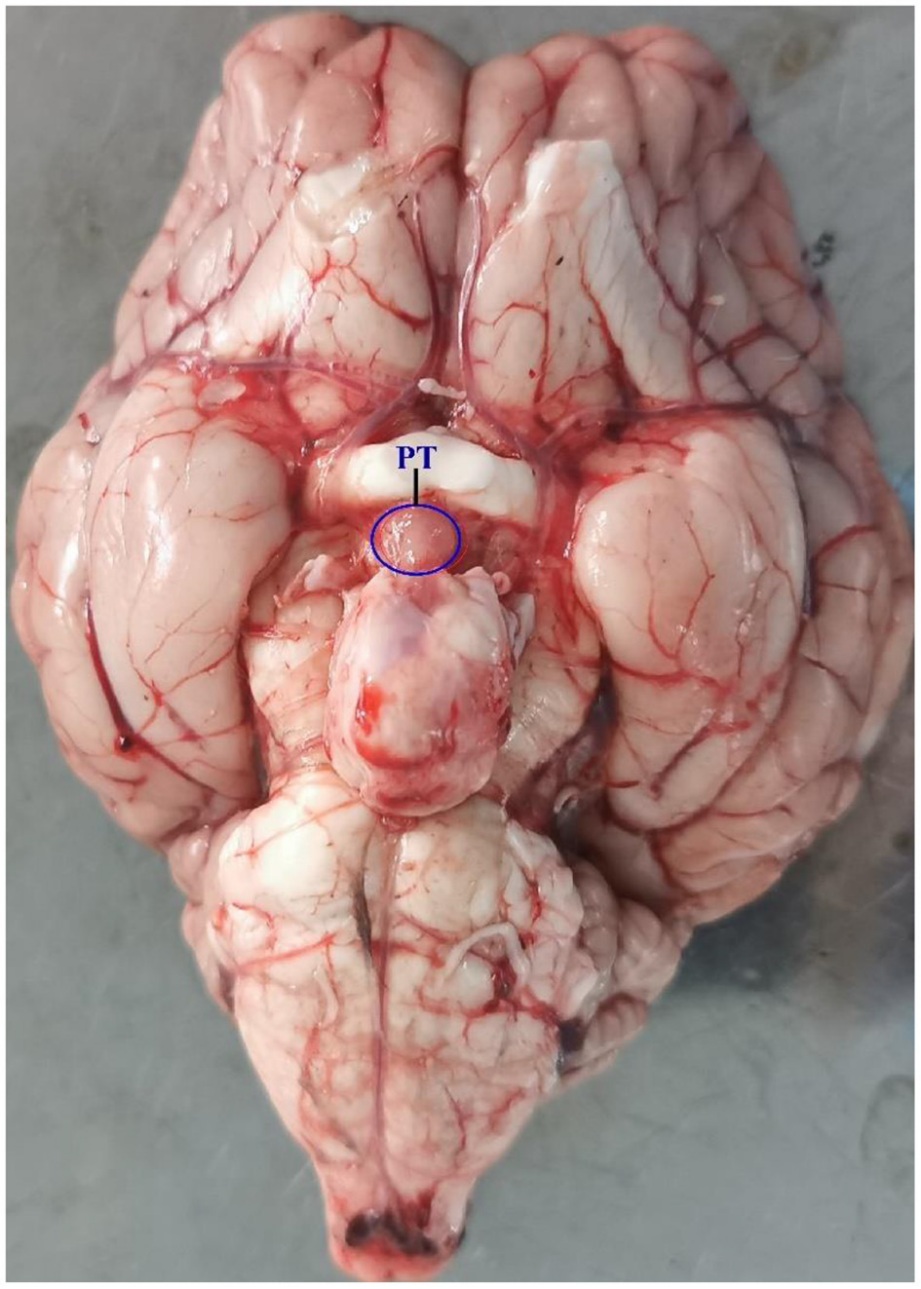
The location of PT tissue in Sunite sheep.

Experiments were conducted on 12 adult Sunite ewes (2–3 years old; weight 30–40 kg), which were selected from a farm in Urat Middle Banner (40° 75′ north latitude), Bayan Nur City, Inner Mongolia Autonomous Region, China, and maintained in a farm in the Tianjin Institute of Animal Sciences, Tianjin (39° 13′ north latitude), China. All ewes were raised under the same conditions, with free access to water and feed. Construction of ovariectomized (OVX) and estradiol-implanted sheep model and light control experiment were previously described in detail (2, 12). Ewes were ovariectomized and estradiol-implanted (E2, Sigma Chemical Co., St. Louis, MO, USA) to maintain plasma estradiol levels of 3–5 pg/ml (6, 16) in October, 2016, according to the model developed by Karsch et al. (1984) (17). This OVX + E2 model normalizes the level of circulating E2 (12), which uncovers the well-documented central seasonal shift in the negative feedback action of E2 on gonadotropin secretion (18). After the surgery, the ewes recovered for 30 days before artificial light control. Then, in November 2016, all ewes were brought indoors and submitted to a light program simulating the outdoor photoperiodic condition by time-control switch. Firstly, all ewes were kept in artificial SP with lights on during 10:30–18:30 (SP, 8:16 h light/dark) for 21 days and switched to LP with lights on during 06:30–22:30 (LP, 16:8 h light/dark) for 42 days, with free access to water and food. Ewes were euthanized [intravenous pentobarbital (100 mg/kg)] at ZT4 [4 h after lights on] of SP21, LP7, LP21, and LP42 (8, 19–21). Consistent with the specific location described by Wood et al. (7) and Lomet et al. (6), the PT tissue (Figure 1) of each ewe was immediately collected and stored at −80°C for total RNA extraction.

### RNA Extraction, Library Construction, and Sequencing

Pituitary PT tissues were used for RNA extraction with TRIzol Reagent (Invitrogen, Carlsbad, CA, USA) according to the manufacturer's instruction. Examination involving the integrality and quality of the isolated RNA was performed via electrophoresis and the RNA Nano 6000 Assay Kit of the Bioanalyzer 2100 system (Agilent Technologies, Santa Clara, CA, USA).

The rRNA was depleted from 3 μg of total RNA using Ribo-Zero TM Gold Kits (Epicentre, Madison, WI, USA). Sequencing libraries of the 12 samples (SP21, *n* = 3; LP7, *n* = 3; LP21, *n* = 3; LP42, *n* = 3) were generated using NEB Next Ultra Directional RNA LibraryPrep Kit for Illumina (NEB, Ipswich, MA, USA) according to the manufacturer's instructions, and index codes were used to label the sequences of each sample. After cluster generation, the library preparations were sequenced on an Illumina Hiseq platform (Illumina, San Diego, CA, USA). Raw data of the performed RNA-seq have been recorded in the SRA public database (Accession number of BioProject: PRJNA680667).

### Reference Genome Mapping and Transcriptome Assembly

Raw data in fastq format were processed through in-house perl scripts. In this step, clean reads were obtained by removing reads with adapter contamination, reads that contained poly-N, and low-quality reads from raw data. Simultaneously, the Q20, Q30, and GC contents of the clean data were calculated. All downstream analysis was based on high-quality clean data. HiSAT2 (22) was used to align clean reads of each sample to the sheep reference genome *Oar_v3.1*. StringTie (23) was used for transcriptome assembly and reconstruction. Thus, known lncRNA and mRNA transcripts were identified, and the position of transcripts was obtained.

### LncRNA Identification and Differentially Expression Analysis

Novel lncRNAs with more than two exons and lengths of more than 200 nt were predicted by CNCI (24), CPC (25), PFAM (26), and CPAT (27) software after transcriptome assembly. The fragments per kilobase per million mapped reads [FPKM (28)] values were calculated to represent the expression of genes and lncRNAs. To determine the effect of photoperiod on genes and lncRNAs expression in PT of sheep, the expression of genes and lncRNAs at every time point of LP (LP7, LP 21, or LP 42) was compared with the SP21 group using DESeq, i.e., SP 21 vs. LP 7, SP 21 vs. LP 21, and SP 21 vs. LP 42. In addition, *p* < 0.05 and |Fold change| >1 was considered standard of differential expression between the SP and LP.

### GO and KEGG Pathway Enrichment Analysis of Differentially Expressed Genes and lncRNAs

Gene Ontology (GO) enrichment analysis of differentially expressed genes or lncRNA target genes was implemented by the GOseq R package, in which gene length bias was corrected (29). GO classifies functions into three groups: cellular components, molecular functions, and biological processes. The KEGG biological pathways database (http://www.genome.jp) is a central public database for understanding high-level functions and regulatory network research. Enrichment analysis was performed on each pathway in KEGG using a hypergeometric test. According to significant threshold (*p*-value: 0.05), the genes were screened and enriched for the pathways. Next, the significance of the pathway enrichment analysis was corrected by FDR, and the corrected *p*-value (*q*-value) was obtained.

### Construction of Integral lncRNA–mRNA Interaction Networks

The primary role of lncRNAs, which are a type of non-coding RNA, is to regulate their target genes by cis-regulating nearby protein-coding genes and trans-regulating distal protein-coding genes. Here, protein coding genes with a distance of <100 Kb were assumed to be the cis-target genes, and Pearson correlation coefficients with the lncRNAs of >0.95 were assumed to represent the trans-target genes (30).

To further reveal the potential roles of lncRNAs that are involved in modulating the reproductive process, integral interaction networks containing lncRNAs and their corresponding target genes were built using Cytoscape software (31) in each comparison (SP 21 vs. LP 7, SP 21 vs. LP 21, and SP 21 vs. LP 42), which include cis- and trans-forms of regulation.

### RNA-seq Data Validation

*EYA3, TSHB, CHGA, MSTRG.209166, MSTRG.22781*, and *MSTRG.42035* were selected to validate the accuracy of RNA sequencing *via* the reverse-transcription quantitative polymerase chain reaction (RT-qPCR). *GAPDH* was used as an internal reference to normalize target gene expression. All primers used in the RT-qPCR are shown in Supplementary Table 1. cDNA was used to perform RT-qPCR after reverse transcription from total RNA to cDNA. The RT-qPCR reaction conditions were as follows: 95°C for 15 min, followed by 40 cycles of 95°C for 10 s and 60°C for 30 s. The data obtained from RT-qPCR reaction were then calculated using the 2^−ΔΔCt^ method (32, 33) and processed by SPSS 19.0 with a one-way analysis of variance. The results are presented as means ± standard deviation. Furthermore, *p* < 0.05 was regarded as statistically significant.

## Results

### Summary of Transcriptome Sequencing Data

To identify differentially expressed lncRNAs and genes between SP and LP, RNA libraries of different photoperiods were constructed. After removing low-quality sequences, a total of 1,394,924,578 clean reads with >92.52% of Q30 were obtained after sequencing all 12 libraries. Approximately 92–95% of the reads were successfully aligned to the *Ovis aries* reference genome (*Oar_v3.1*) (Table 1).

**Table 1:** Summary of raw reads after quality control and mapping to the reference genome.

**Photoperiod**	**Sample**	**Raw reads**	**Clean reads**	**Clean reads rate (%)**	**Q30 (%)**	**Mapped reads**	**Mapping rate (%)**
SP21	SP2101	125,660,438	122,442,196	97.44	96.91	115,418,878	94.26
	SP2102	125,942,826	122,243,686	97.06	95.9	114,892,152	93.99
	SP2103	132,973,810	129,237,768	97.19	95.94	121,797,173	94.24
LP7	LP701	113,017,724	109,422,884	96.82	94.45	102,336,864	93.52
	LP702	120,666,156	116,544,984	96.58	94.44	108,968,477	93.50
	LP703	120,732,238	117,373,382	97.22	96.09	110,710,654	94.32
LP21	LP2101	109,242,794	106,213,878	97.23	93.56	98,166,189	92.42
	LP2102	123,453,896	119,838,276	97.07	96.16	113,659,315	94.84
	LP2103	122,212,386	116,562,600	95.38	92.52	108,893,984	93.42
LP42	LP4201	112,488,634	109,485,312	97.33	93.83	101,355,097	92.57
	LP4202	108,789,602	105,051,072	96.56	94.51	98,080,693	93.36
	LP4203	123,811,728	120,508,540	97.33	95.94	113,375,002	94.08

*SP, short photoperiod; LP, long photoperiod; Q30, the rate of bases whose quality is >30 Phred value in clean reads*.

**Figure 2:**
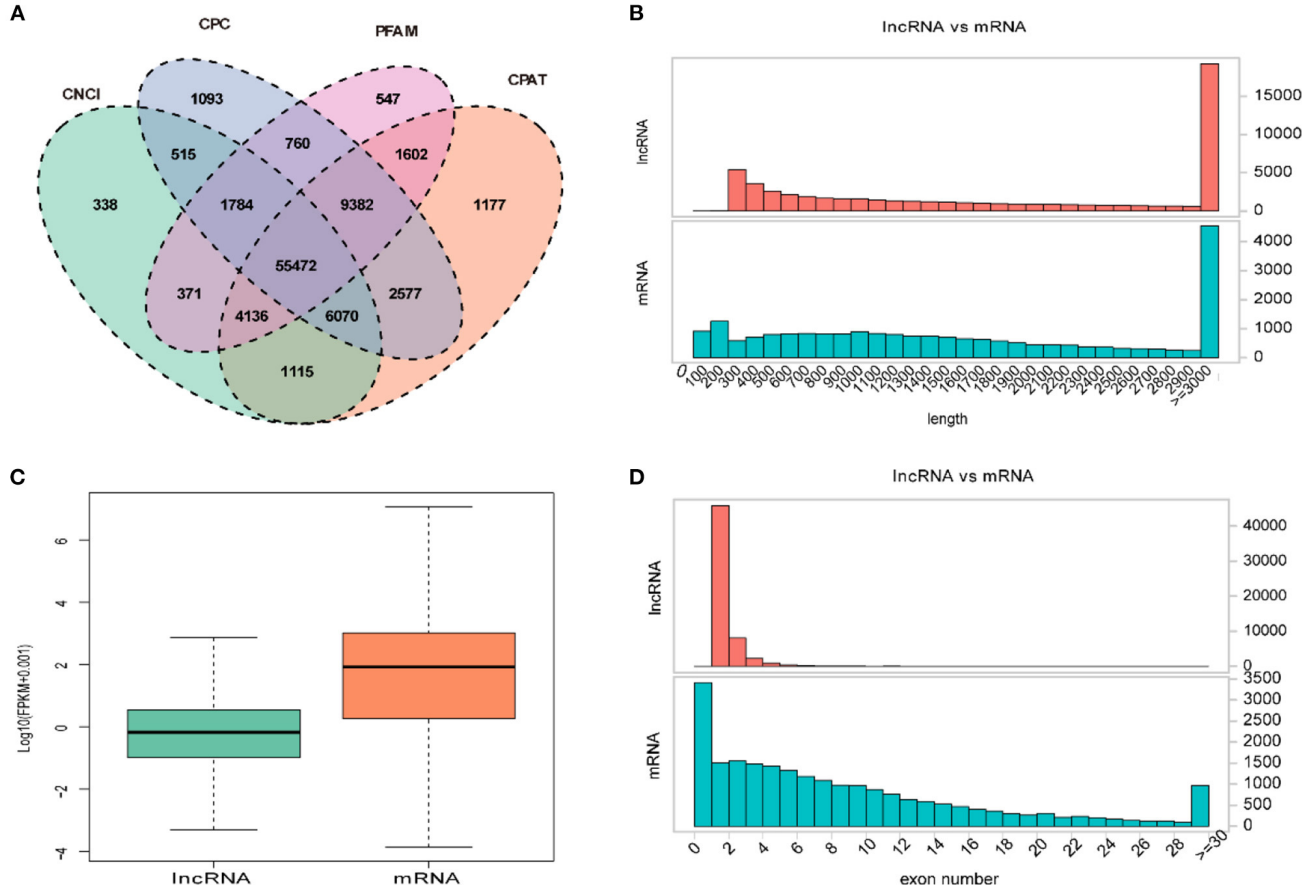
Expression characteristics of lncRNAs and genes in the PT of ewes. **(A)** Venn diagram for screening lncRNAs by four software (CNCI, CPC, CAPT, and PFAM). The sum of the numbers in each large oval represents the total number of non-coding transcripts from each software, and the overlapping parts of the oval represent the non-coding transcripts common to the software. **(B)** The length distribution of lncRNAs and mRNAs. **(C)** The expression level of lncRNAs and mRNAs. **(D)** The exon number distribution of lncRNAs and mRNAs.

Overall, a total of 55,472 lncRNAs were identified in PT of 12 ewes using four programs (CNCI, CPC, PFAM, and CAPT) (Figure 2A). The length distribution of lncRNAs was consistent with that of protein-coding gene (Figure 2B). Of them, the transcripts of lncRNAs and mRNAs with lengths of more than 3,000 bp accounted for the majority, and its number in lncRNAs was significantly greater than that in mRNAs (Figure 2B). However, transcript levels of lncRNAs were lower than those of mRNAs in the PT of Sunite ewes (Figure 2C). Most of the lncRNAs have only two or three exons, whereas mRNAs contain a wide range of exons from 2 to 30 (Figure 2D).

### Photoperiod-Induced Factor Analysis Through Differentially Expressed Genes and lncRNAs

**Figure 3:**
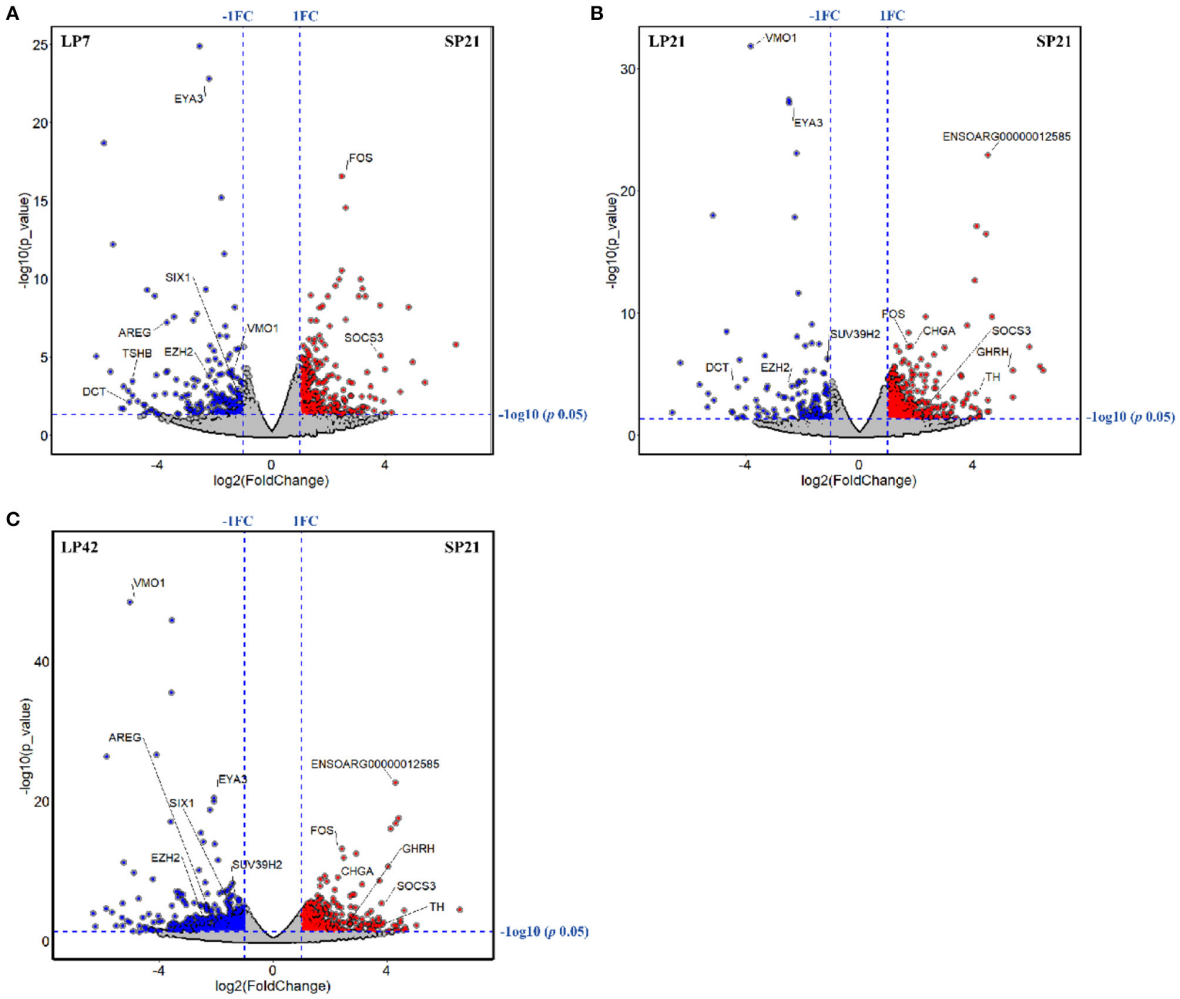
The volcano plots of differentially expressed genes between long photoperiod (LP) and short photoperiod (SP) in PT of Sunite sheep. **(A)** The results of LP7 vs. SP21; **(B)** the results of LP21 vs. SP21; **(C)** the results of LP42 vs. SP21.

The number of differentially expressed (DE) genes between SP and LP increased gradually with the extension of LP (from LP7 to LP42). Specifically, there were 499 DE mRNAs in SP21 vs. LP7, in which *FOS, SOCS3, EYA3, TSHB, SIX1, DCT, VMO1, AREG*, and *EZH2* were related to reproduction (Figure 3A). In SP21 vs. LP21, 625 DE mRNAs were detected, including *CHGA, FOS, SOCS3, GHRH, TH, EYA3, TSHB, DCT, VMO1, SUV39H2*, and *EZH2* that were associated with reproduction (Figure 3B). In addition, 874 DE mRNAs were screened between the SP21 and LP42 group. Of them, the genes related to reproduction included *CHGA, FOS, SOCS3, GHRH, TH, EYA3, TSHB, SIX1, VMO1, AREG*, and *EZH2* (Figure 3C). The top 10 DE mRNAs in each contrast group are shown in Table 2. Among these genes, there are specific differentially expressed genes (*FOS, TMEM200A, MLIP, PMP2, PRX*, and *SPTBN5* and seven novel genes) that are different from previous reports (6, 7). Besides, for the top genes, it is worth paying attention to those genes that appear in the above contrast groups simultaneously, and they are the most likely photoperiodic-induced factors in the regulation of seasonal reproduction. To sum up, the notable LP-induced genes included *EYA3, TSHB, VMO1, DCT*, and *EZH2*. SP-induced genes involved *ENSOARG00000012585, CHGA, FOS, SOCS3*, and *TH*. For lncRNAs, the number of DE lncRNAs was also increasing with the extension of the LP. Specifically, 1,887, 2,038, and 2,614 DE lncRNAs were detected in the SP21 vs. LP7 group (Supplementary Table 5), SP21 vs. LP21 group (Supplementary Table 6), and SP21 vs. LP42 group (Supplementary Table 7), respectively. These DE lncRNAs may be involved in the regulation of the expression of important genes for seasonal reproductive trait. Besides, our results had some common overlap DE mRNAs with the reports of Wood et al. (2015) and Lomet et al. (2017) (6, 7). The detailed information is shown in Supplementary Table 8.

**Table 2:** The top 10 differentially expressed mRNAs in three comparisons between LP and SP.

**Comparisons**	**Gene**	***p*-value**	**Log2foldchange**	**Up/Down**	**Gene name**
LP7 vs. SP21	OARG00000014847	1.29E−25	−2.531934183	Up	*ENSOARG00000014847*
	OARG00000003071	1.55E−23	−2.209131416	Up	*EYA3*
	OARG00000002985	2.10E−19	−5.881442781	Up	*ENSOARG0000000298*
	OARG00000001783	2.83E−17	2.460057284	Down	*FOS*
	OARG00000017954	6.56E−16	−1.77693187	Up	*ACSL4*
	OARG00000012585	2.89E−15	2.610496836	Down	*ENSOARG00000012585*
	OARG00000015485	6.34E−13	−5.568818193	Up	*DQA*
	OARG00000020015	2.54E−12	−1.658620427	Up	*TMEM200A*
	OARG00000000857	3.00E−11	2.448160683	Down	*ENSOARG00000000857*
	OARG00000003776	1.04E−10	3.130953431	Down	*ENSOARG00000003776*
LP21 vs. SP21	OARG00000007112	1.47E−32	−3.819122926	Up	*VMO1*
	OARG00000005662	9.30E−29	−9.037863894	Up	*ENSOARG00000005662*
	OARG00000017954	3.92E−28	−2.474582452	Up	*ACSL4*
	OARG00000003071	6.86E−28	−2.454903078	Up	*EYA3*
	OARG00000008195	8.26E−24	−2.196263967	Up	*CYP4V2*
	OARG00000012585	1.20E−23	4.510495819	Down	*ENSOARG00000012585*
	OARG00000018081	1.59E−23	−8.570717405	Up	*BPIFC*
	OARG00000008250	1.05E−18	−5.150050353	Up	*KLKB1*
	OARG00000014847	1.50E−18	−2.278049468	Up	*ENSOARG00000014847*
	OARG00000009143	7.84E−18	4.11635763	Down	*ENSOARG00000009143*
LP42 vs. SP21	OARG00000009116	2.03E−56	−7.230483397	Up	*PMP2*
	OARG00000007112	2.98E−49	−5.030524117	Up	*VMO1*
	OARG00000017954	1.45E−46	−3.565896967	Up	*ACSL4*
	OARG00000006792	2.83E−43	−7.98187898	Up	*MLIP*
	OARG00000006630	3.02E−36	−3.574968045	Up	*PRX*
	OARG00000020448	2.35E−27	−4.099272015	Up	*SPTBN5*
	OARG00000008250	4.24E−27	−5.842338208	Up	*KLKB1*
	OARG00000018081	1.34E−23	−8.659205306	Up	*BPIFC*
	OARG00000012585	2.18E−23	4.291991999	Down	*ENSOARG00000012585*
	OARG00000003071	3.48E−21	−2.091523071	Up	*EYA3*

### GO Annotation and KEGG Enrichment Analysis of DE Genes and lncRNAs

**Figure 4:**
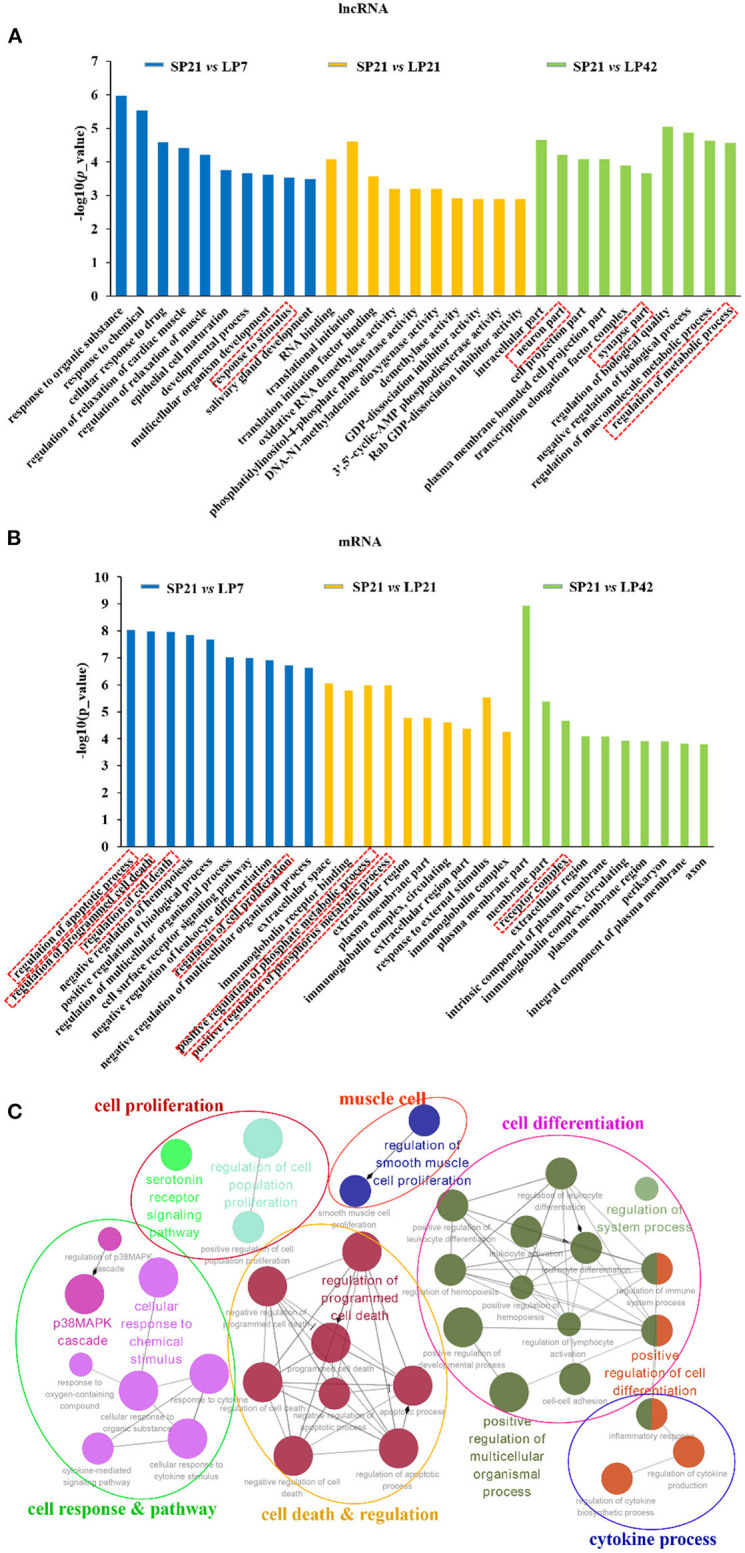
Top enriched Gene Ontology (GO) terms of differentially expressed lncRNAs and genes between short photoperiod and long photoperiod in PT of Sunite sheep. **(A)** The top GO terms of target genes of differentially expressed LncRNAs; **(B)** the top GO terms of differentially expressed mRNAs. The GO terms in blue are from SP21 vs. LP7; those in orange are from SP21 vs. LP21, and those in green are from SP21 vs. LP42. **(C)** A simplified network of related statistically significant GO terms (*p* < 0.01) using the Cytoscape add-on ClueGO (34, 35). The network of overlap DE genes among SP21 vs. LP7, LP21, and LP42 are shown. The filled colored circles (nodes) represent each statistically significant parent GO term. The lines (edges) between the nodes show that there are overlapping genes within each term. The colored ovals group these parent GO terms into more generic functional descriptions. The red boxes represent the seasonal reproduction-related terms.

GO and KEGG analyses were conducted for the differentially expressed mRNAs and target genes of differentially expressed lncRNAs. The top 10 enriched GO terms for lncRNAs and mRNAs of SP21 vs. LP7 (blue), SP21 vs. LP21 (orange), and SP21 vs. LP42 (green) are shown in Figures 4A,B (data in Supplementary Tables 9–11 for lncRNAs and Supplementary Tables 12–14 for mRNAs). The following GO terms are noteworthy, including neuron terms (such as neuron part, axon, synapse part, and response to stimulus), receptor terms (such as cell surface receptor signaling pathway and receptor complex), regulation of apoptosis process, regulation of cell death, regulation of cell proliferation, and metabolic process (such as regulation of metabolic process and positive regulation of phosphorus metabolic process). In order to further focus on the function of differentially expressed genes between SP and LP, we constructed a simplified network of related statistically significant GO terms (Figure 4C) for differentially expressed genes overlapping between SP and three of LP points using the Cytoscape add-on ClueGO (34, 35). The results emphasized enrichment for cell proliferation and differentiation, cell response and pathway, cell death and regulation, and the cytokine process, which might play an important role in the regulation of seasonal breeding in sheep at the level of the PT/MBH (36, 37).

**Figure 5:**
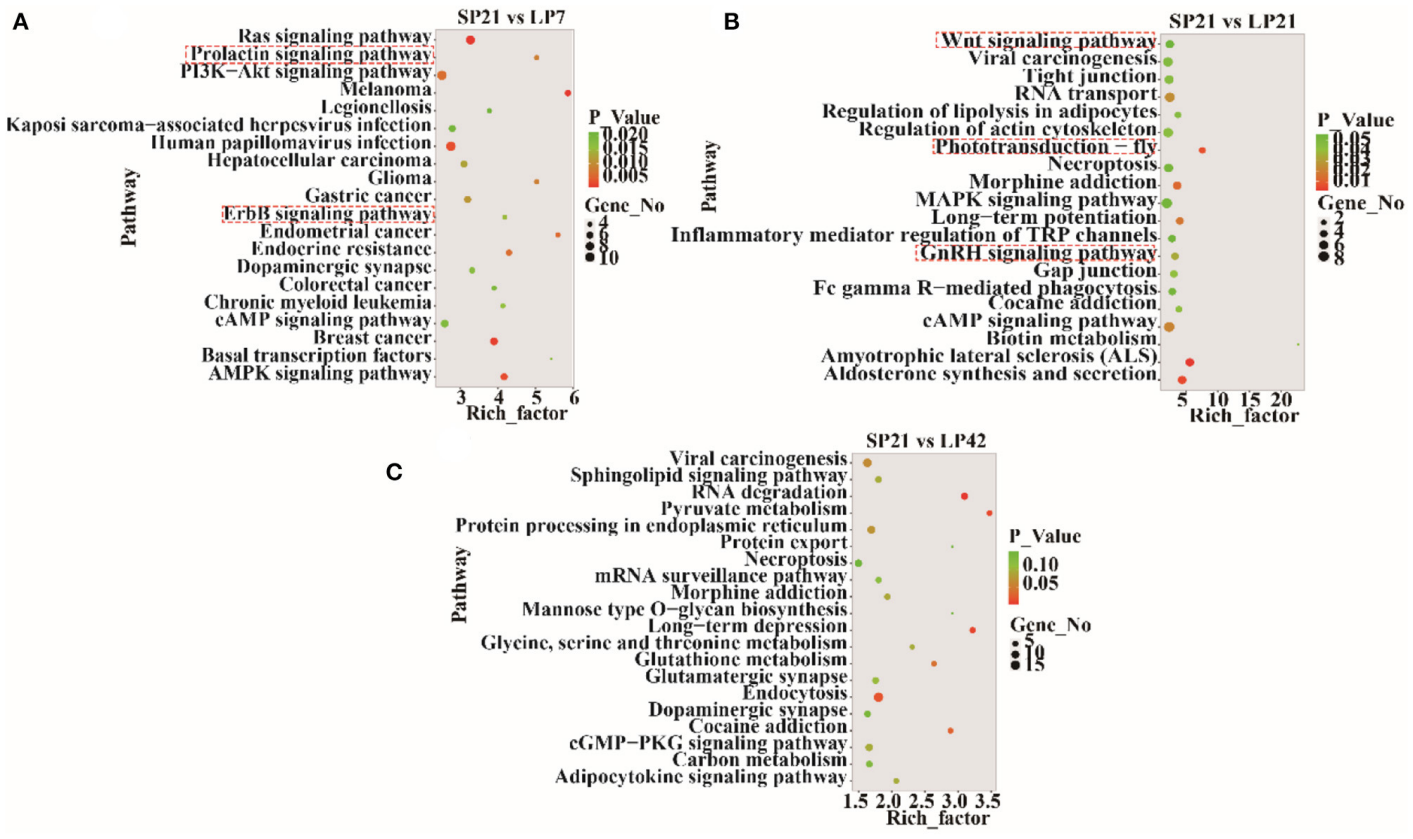
KEGG pathway analysis for target genes of differentially expressed lncRNAs between short photoperiod and long photoperiod in PT of Sunite sheep. **(A)** Top 20 enrichment pathways in SP21 vs. LP7. **(B)** Top 20 enrichment pathways in SP21 vs. LP21. **(C)** Top 20 enrichment pathways in SP21 vs. LP42. Rich_factor is defined as the amount of differentially expressed genes enriched in the pathway/amount of all genes in the background gene set. The red boxes represent the seasonal reproduction-related terms.

**Figure 6:**
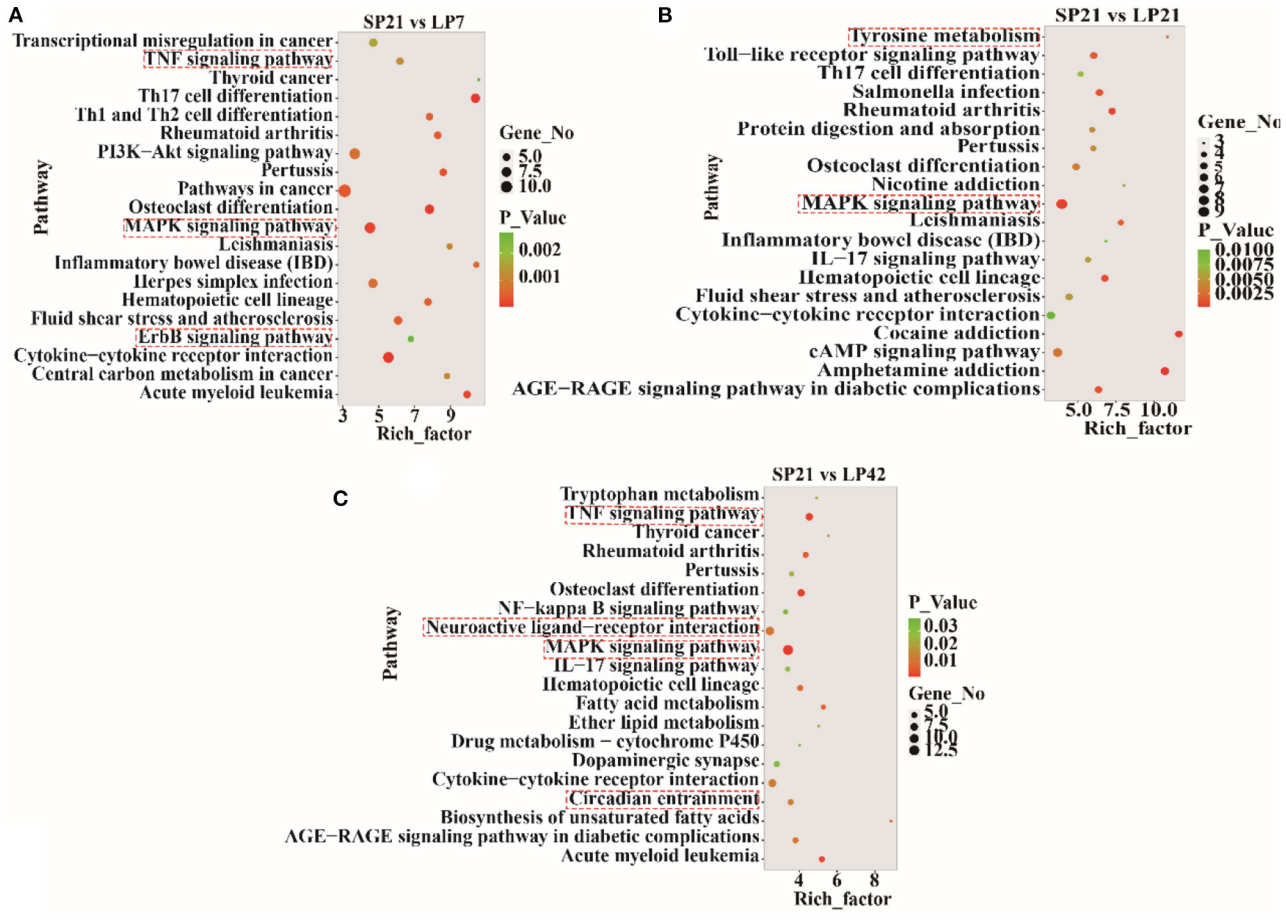
KEGG pathway analysis of differentially expressed mRNAs between short photoperiod and long photoperiod in PT of Sunite sheep. **(A)** Top 20 enrichment pathways in SP21 vs. LP7. **(B)** Top 20 enrichment pathways in SP21 vs. LP21. **(C)** Top 20 enrichment pathways in SP21 vs. LP42. Rich_factor is defined as the amount of differentially expressed genes enriched in the pathway/amount of all genes in the background gene set. The red boxes represent the seasonal reproduction-related terms.

**Figure 7:**
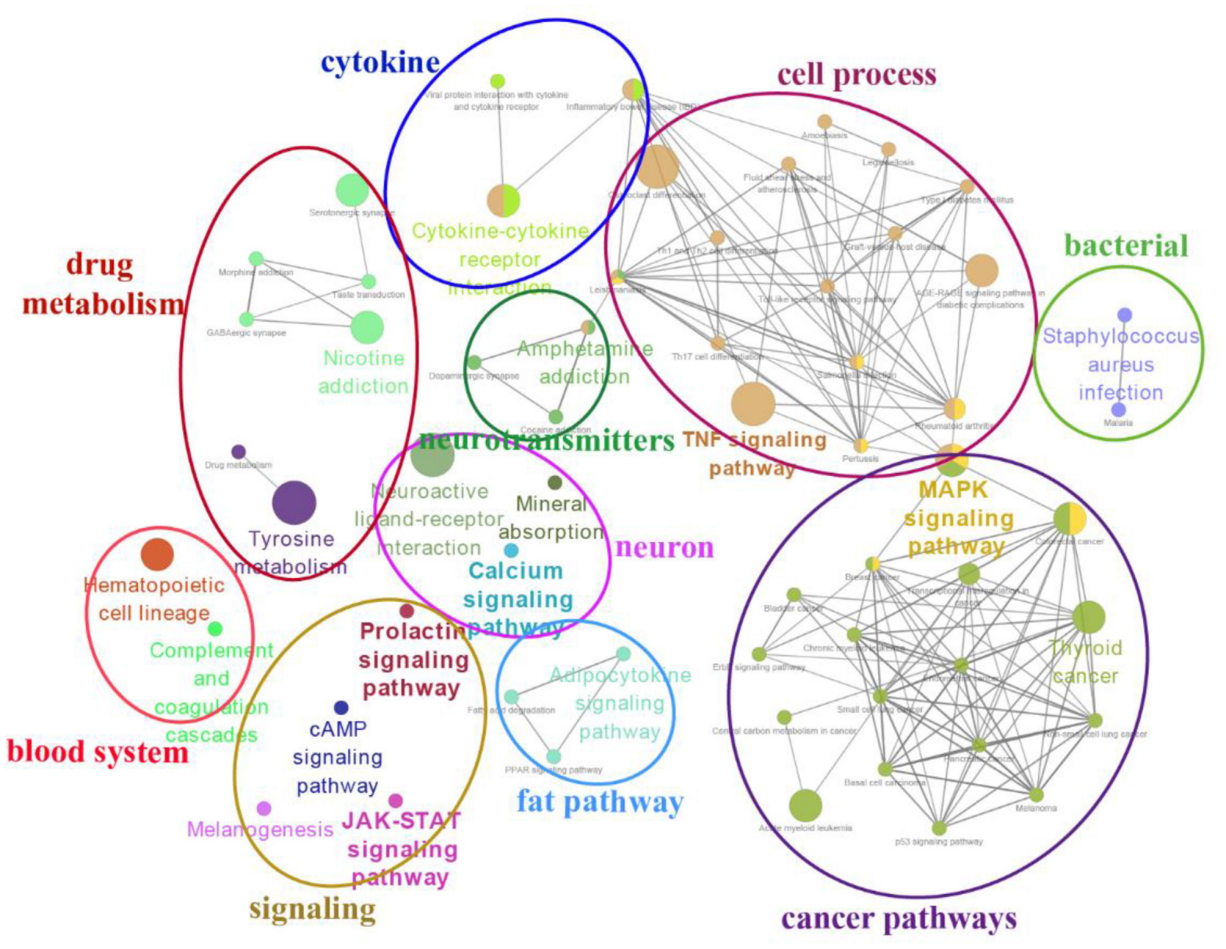
A simplified network of related statistically KEGG pathways using the Cytoscape add-on ClueGO (34, 35). The network of overlap DE genes among SP21 vs. LP7, LP21, and LP42 are shown. The filled colored circles (nodes) represent each statistically parent KEGG pathway. The lines (edges) between the nodes show that there are overlapping genes within each pathway. The colored ovals group these parent KEGG pathways into more generic functional descriptions.

The top 20 enriched KEGG pathways for target genes of differentially expressed lncRNAs and pathways for differentially expressed mRNAs are shown in Figures 5, 6, respectively. The most interesting pathways included reproduction-related pathways (Prolactin signaling pathway, ErbB signaling pathway, Wnt signaling pathway, MAPK signaling pathway, and GnRH signaling pathway in Figure 5; ErbB signaling pathway and MAPK signaling pathway in Figure 6), circadian related pathways (phototransduction-fly in Figure 5; circadian entrainment in Figure 6), and neuroactive ligand–receptor interaction, TNF signaling pathway, tyrosine metabolism, and neuroactive ligand–receptor interaction pathway (detailed data in Supplementary Tables 15–17 for lncRNAs and Supplementary Tables 18–20 for mRNAs). Here, we also combined the overlap DE genes from three comparisons and assigned KEGG terms to create a simplified network of related statistically significant KEGG terms (34, 35) (Figure 7). Figure 7 also showed that many differentially expressed genes between SP and LP are enriched in pathways related to reproduction, such as signaling (prolactin signaling pathway, cAMP signaling pathway, and JAK-STAT signaling pathway), neuron, neurotransmitters, and cell process.

### LncRNA–mRNA Network Construction

**Figure 8:**
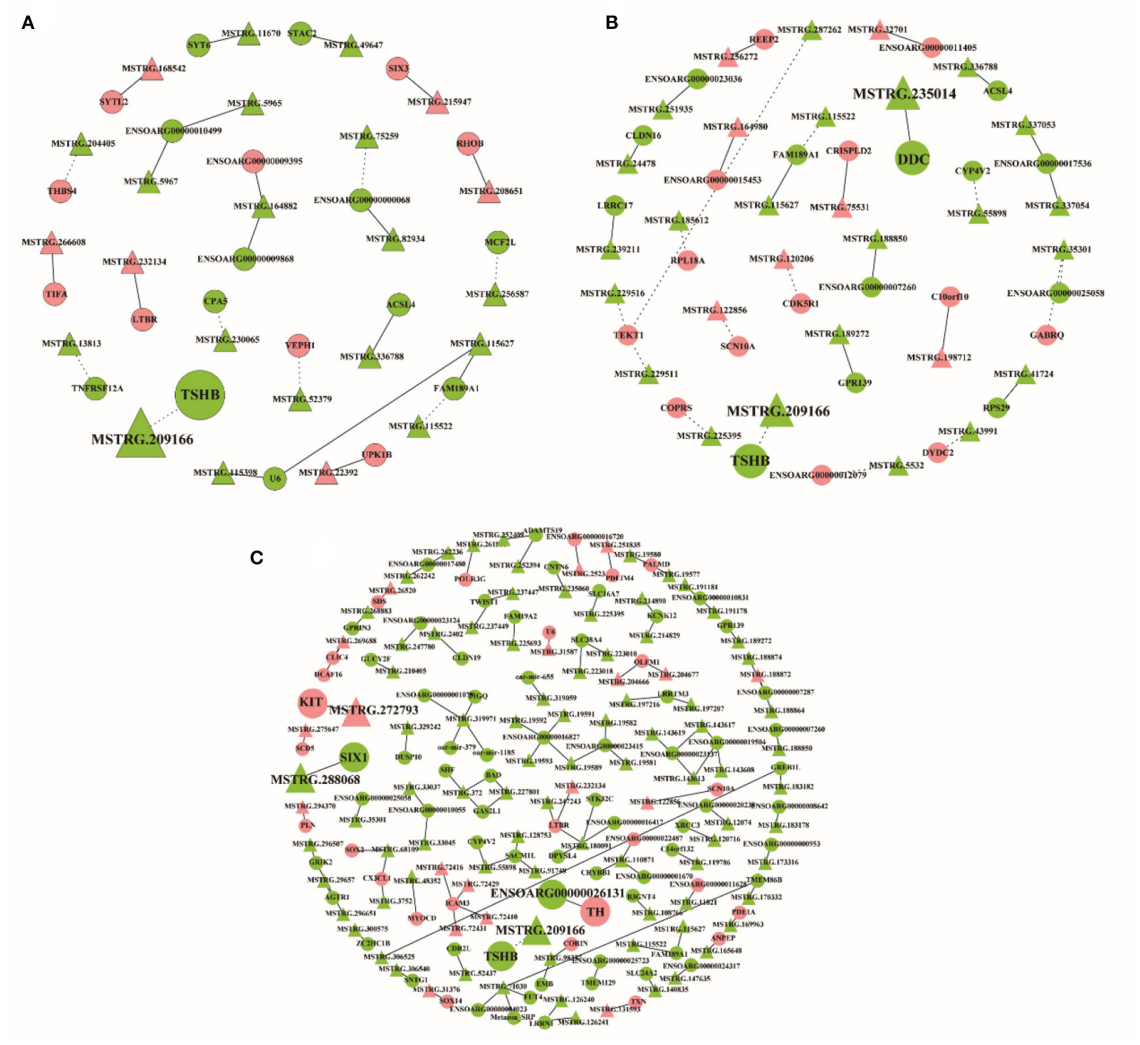
The interaction networks of lncRNAs and their corresponding target gene. **(A)** The networks for SP21 vs. LP7; **(B)** the networks for SP21 vs. LP21; **(C)** the networks for SP21 vs. LP42. The dashed and solid lines represent trans- and cis-regulation functions, respectively. Red and green represent up- and downregulation, respectively. Circles and triangles represent mRNAs and lncRNAs, respectively.

The differentially expressed lncRNAs and their target genes were selected to construct the lncRNA–mRNA network with co-expression information. In the network of SP21 vs. LP7, 23 differentially expressed lncRNAs and 21 target genes were included, among which the *trans* relationship between *MSTRG.209166* and *TSHB* was hinted (Figure 8A, Supplementary Table 21). In SP21 vs. LP21, 29 differentially expressed lncRNAs and 26 target genes were selected to construct network. Notably, *MSTRG.209166* was also found to *trans*-regulate *TSHB* and *MSTRG.235014 cis*-regulated *DDC*, because the two genes were important for seasonal reproduction (Figure 8B, Supplementary Table 22). In SP21 vs. LP42, 102 differentially expressed lncRNAs and 91 target genes formed a more sophisticated network. For several key genes of seasonal reproduction, several target combinations between lncRNAs and them were highlighted. For example, they implied that *MSTRG.209166 trans*-regulated *TSHB, MSTRG.288068 cis-*regulated *SIX1, MSTRG.272793 cis*-regulated *KIT*, and *ENSOARG00000026131 cis*-regulated *TH* (Figure 8C, Supplementary Table 23).

### Gene Expression Validation

**Figure 9:**
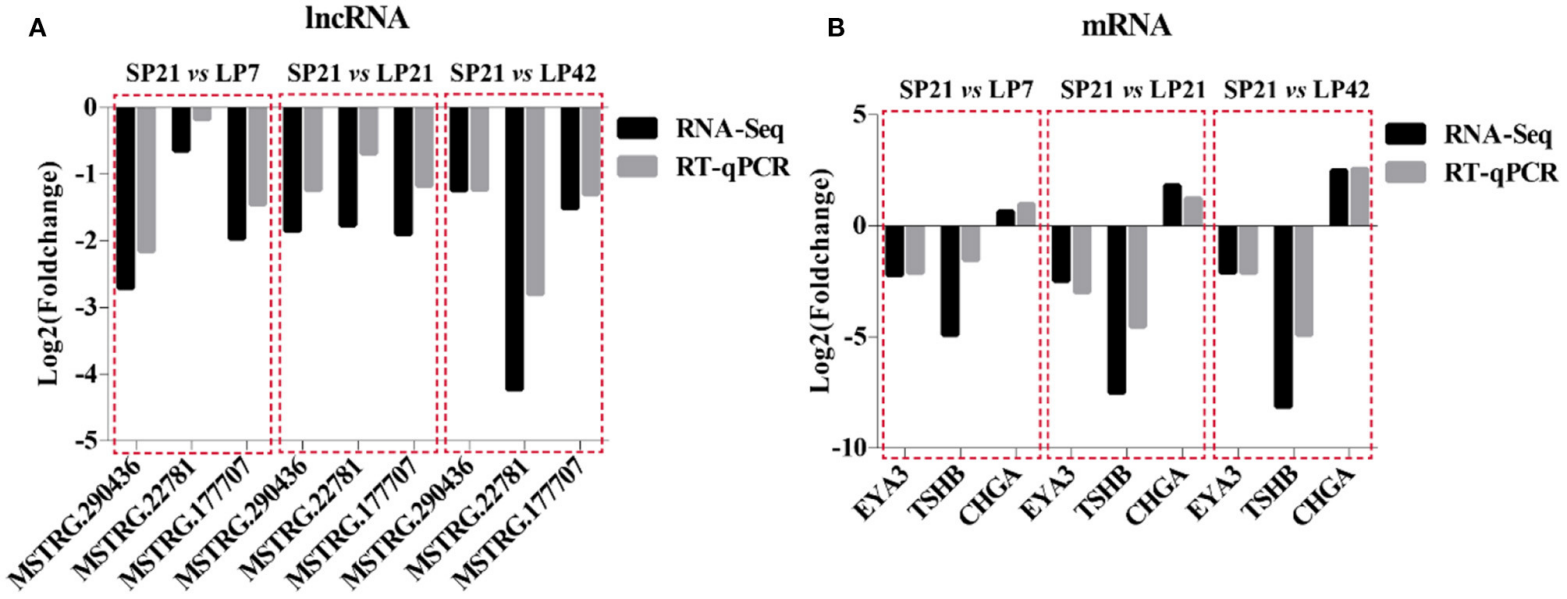
Validation of RNA-Sequencing (RNA-seq) data using RT-qPCR. **(A)** RNA-Seq and RT-qPCR results of three selected differentially expressed lncRNAs in PT of Sunite sheep at different photoperiods. **(B)** RNA-Seq and RT-qPCR results of three selected differentially expressed mRNAs in PT of Sunite sheep at different photoperiods.

A total of six genes, namely, three mRNAs (*EYA3, TSHB*, and *CHGA*) related to seasonal reproduction and three random lncRNAs (*MSTRG.290436, MSTRG.22781*, and *MSTRG.17707*), were selected for RT-qPCR verification. The results indicated that there is a similar expression pattern between RNA-Seq and RT-qPCR data (Figure 9).

## Discussion

Seasonal reproduction is a result of adaptation of animal reproductive activities to environmental changes, which is essential for breeding success and survival of future generations. However, the detailed molecular mechanism of animal seasonal reproduction has not been fully revealed. So far, several key genes for seasonal reproduction have been found in animals, including *EYA3* gene in Japanese quail (38) and sheep (8), the vasoactive intestinal peptide (*VIP*) gene in Yangzhou goose (39), *TSHB* in sheep (8), *TSHB* and pituitary adenylate cyclase activating polypeptide (*PACAP*) in mice (19, 40), and *CHGA* and Tachykinin 1 (*TAC1*) in sheep (3, 7), which are mainly expressed in the pituitary PT. The pituitary PT is an important regulatory center for seasonal reproductive traits. Therefore, it was used as the target tissue in this study and the first objective of this study is to screen new candidate photoperiod-induced genes in PT of sheep.

Combined with the results of gene differential expression, GO, and KEGG pathway enrichment analyses, some important candidate genes were screened out. They included *CHGA, FOS, SOCS3, EYA3, TSHB, SIX1, GHRH, DCT, TH, VMO1, AREG, SUV39H2*, and *EZH2*, which were mainly involved in the following pathways: reproduction-related pathways, TNF signaling pathway, circadian entrainment, and tyrosine metabolism. Specifically, LP-induced genes included *EYA3, VMO1, TSHB, SIX1, GHRH, DCT, TH, VMO1, AREG, SUV39H2*, and *EZH2* and SP-induced genes involved *ENSOARG00000012585, CHGA, FOS, SOCS3*, and *TH*. Wood et al. (7) reported *CHGA* as a SP-activator, which had a significantly high expression at SP compared with LP in PT of rams. Our results in PT of ewes also further confirm that *CHGA* is a SP-induced gene. For *FOS, SOCS3*, and *ENSOARG00000012585*, they may be considered as new candidate SP-induced genes in PT, whose function in PT need further analysis. The suprachiasmatic nuclei of the hypothalamus (SCN) are the master circadian clock in mammals. The role of *FOS* and *SOCS3* in SCN has been reported in long-day breeding animals (redheaded bunting, rat, and hamster), which are enriched in the TNF signaling pathway and circadian entrainment. Their expression patterns under SP and LP are opposite to those of short-day breeding animals (41–43). For example, *FOS* is predominantly expressed in the SCN of redheaded buntings under LP conditions (42). Moreover, our results showed that *FOS* was highly expressed in pituitary PT of sheep under SP. Photoperiod also modulated *SOCS3* gene expression and maintained its expression in a high level in the SCN of hamsters during LP compared with SP (44). Then, *SOCS3* conveys seasonal changes into leptin sensitivity in the Siberian hamster (44).

So far, several LP-induced genes have been revealed. *EYA3* and *TSHB*, as LP-induced genes, displayed a marked increase from SP to LP in PT of sheep (3, 7, 45). Similarly, in this study, *EYA3* and *TSHB* were LP-induced and went up at LP compared with SP in PT of ewes, further certifying the findings of previous studies (8, 15, 46). In both sheep and mouse, *EYA3* and its partner *SIX1* synergistically act as upstream inducers of the *TSHB* transcription to induce the expression of *TSHB* (8, 19). Furthermore, *TSHB* acts locally on *TSHR*-expressing cells in the adjacent basal hypothalamus, leading to altered expression of *DIO2* (5, 6, 47). Then, *DIO2* can regulate the secretion of TH in the basal hypothalamus and further modulate the transition of reproductive status through the spatial structure of GnRH neurons (7). When the GnRH neurons were wrapped by ependymal cells, little GnRH were released into pituitary, which caused the decrease of LH and FSH secretion, and eventually sheep entered the state of anestrus (7).

Tyrosine metabolism can mediate the biological effects of many hormones and cytokines on reproduction, immunity, and cell growth (48, 49). In this study, the DE *DCT* was enriched in the tyrosine metabolism pathway. *DCT* was found as a novel long-day marker, whose expression levels in tanycytes lining the infra-lateral walls and floor of the ovine third ventricle showed marked increase with the extension of the photoperiod (6, 45). Similarly, in this study, *DCT* was significantly upregulated during LP compared with SP in PT of sheep. These results suggested that *DCT* expression was crucial for initiation of anestrus in sheep. Previous research has shown that *TH* can modulate *DCT* expression (46). *DCT* is a part of, or is driven by, the circannual clock in sheep. Therefore, these characteristics—LP induction and circannual changes in expression—place *DCT* in a list of key genes involved in seasonal timing, which had included *TSHB, EYA3, DIO2*, and *DIO3* (45, 46).

In this study, the expression level of *VMO1, AREG, SUV39H2*, and *EZH2* was also significantly higher during LP than those at SP in PT of sheep. As secreted factors, *VMO1* and *AREG* were found to be acutely induced by LP in MBH of Ile-de-France ewes (6). In polyovular species, the LH-driven signaling can promote oocyte maturation and cumulus expansion by *AREG*. In addition, murine data indicated that LH binding to LHCGR in mural granulosa cells will up-regulate *AREG* (50). However, the function of *AREG* gene in PT is still unknown, and it may be considered as a candidate LP-induced gene to analyze. For two histone methyltransferases genes, *SUV39H2* and *EZH2*, the results about seasonal changes of their expression in the PT are consistent with the hypothesis that epigenetic changes in PT cells are involved in circannual timing (6). Besides, the acute LP responsiveness of most of these markers (*TSHB, VMO1, EZH2*, and *EYA3*) is also in accordance with the findings from Dardente et al. (45) and Lomet et al. (6) in Ile-de-France ewes.

In recent years, studies have indicated that lncRNAs play important roles in many aspects of sheep reproductive regulation, such as fecundity (12, 13), gonadal development (14), and sex hormone response (15). As epigenetic regulators, lncRNAs can regulate the expression of reproduction-related genes. Thus, the other objective of this study is to predict lncRNAs that target key genes for seasonal reproduction in PT of sheep. Firstly, our result showed that the sequence length and exon number of mRNAs and lncRNAs in sheep pituitary have different patterns with those in hypothalamus of sheep (3,448 nt and 2.5 exons) (13). This implies that lncRNAs have tissue-specific characteristics. Then, through lncRNA–mRNA network construction, several notable target combinations between lncRNAs and key genes for seasonal reproduction were predicted. They specifically included the *trans* relationship between *MSTRG.209166* and *TSHB* and *cis* relationships between *MSTRG.235014* and *DDC, MSTRG.272793* and *KIT*, and *ENSOARG00000026131* and *TH*. Importantly, the *trans* relationship between *MSTRG.209166* and *TSHB* was shared among all of the comparison groups (SP vs. LP), which suggested that *MSTRG.209166* might play an important role in seasonal reproduction by regulating the expression of *TSHB*. TSHB is an important hub in the pathway of seasonal reproduction, so this study provides a new molecular object (*MSTRG.209166*) for the follow-up epigenetic regulation study of seasonal reproduction in sheep. In addition, the several candidate lncRNAs mentioned above (e.g., *MSTRG.235014, MSTRG.272793*, and *ENSOARG00000026131*) are also worthy of in-depth analysis.

## Conclusion

In summary, our study provided a genome-wide view of lncRNA and mRNA expression profiling in pituitary PT of sheep during LPs and SPs. Several new candidate photoperiod-induced genes and lncRNAs targeting key genes of seasonal reproduction were predicted in PT of sheep. These results will provide new clues for understanding the molecular regulation of seasonal reproduction in sheep.

## Data Availability Statement

The datasets presented in this study can be found in online repositories. The names of the repository/repositories and accession number(s) can be found below: NCBI BioProject; PRJNA680667.

## Ethics Statement

The animal study was reviewed and approved by The Science Research Department (in charge of animal welfare issue) of the Institute of Animal Sciences, Chinese Academy of Agricultural Sciences (IAS-CAAS; Beijing, China) (No. IAS2018-3,10 April 2018). Written informed consent was obtained from the owners for the participation of their animals in this study.

## Author Contributions

QX and MC: design of experiment and analysis for PT of sheep. QL and XH: construction of the OVX+E2 sheep model and light control experiment. RD: Experimental guidance and writing. XZ, JZ, and XG: light control experiment and collection of samples.

## Conflict of Interest

The authors declare that the research was conducted in the absence of any commercial or financial relationships that could be construed as a potential conflict of interest.

## Publisher's Note

All claims expressed in this article are solely those of the authors and do not necessarily represent those of their affiliated organizations, or those of the publisher, the editors and the reviewers. Any product that may be evaluated in this article, or claim that may be made by its manufacturer, is not guaranteed or endorsed by the publisher.
