# Corrigendum: Identification of Photoperiod-Induced LncRNAs and mRNAs in Pituitary Pars Tuberalis of Sheep

**DOI:** 10.3389/fvets.2021.753614

**Published:** 2021-09-24

**Authors:** Qing Xia, Mingxing Chu, Xiaoyun He, Qiuyue Liu, Xiaosheng Zhang, Jinlong Zhang, Xiaofei Guo, Ran Di

**Affiliations:** ^1^Key Laboratory of Animal Genetics and Breeding and Reproduction of the Ministry of Agriculture and Rural Affairs, Institute of Animal Science, Chinese Academy of Agricultural Sciences, Beijing, China; ^2^Institute of Genetics and Developmental Biology, Chinese Academy of Sciences, Beijing, China; ^3^Tianjin Institute of Animal Sciences, Tianjin, China

**Keywords:** lncRNAs, mRNAs, photoperiod, sheep, pituitary pars tuberalis

Author Qiuyue Liu was not included as an author in the published article. Now the author has been added to this article. The corrected Author Contributions Statement appears below.

QX and MC: design of experiment and analysis for PT of sheep. QL and XH: construction of the OVX+E2 sheep model and light control experiment. RD: Experimental guidance and writing. XZ, JZ, and XG: light control experiment and collection of samples.

In the original article, the reference no. 12 was incorrectly written as La Y, Tang J, He X, Di R, Chu M. Identification and characterization of mRNAs and lncRNAs in the uterus of polytocous and monotocous Small Tail Han sheep (Ovis aries). *PeerJ*. (2019) 7:e6938. doi: 10.7717/peerj.6938. It should be He X, Tao L, Zhong Y, Di R, Xia Q, Wang X, et al. Photoperiod induced the pituitary differential regulation of lncRNAs and mRNAs related to reproduction in sheep. *PeerJ*. (2021) 9:e10953. doi: 10.7717/peerj.10953.

In the original article, there was a sentence missing in the Methods section, subsection “Animal and Tissue Acquirement,” first paragraph. The complete paragraph appears below.

Experiments were conducted on 12 adult Sunite ewes (2–3 years old; weight 30–40 kg), which were selected from a farm in Urat Middle Banner (40° 75′ north latitude), Bayan Nur City, Inner Mongolia Autonomous Region, China, and maintained in a farm in the Tianjin Institute of Animal Sciences, Tianjin (39° 13′ north latitude), China. All ewes were raised under the same conditions, with free access to water and feed. Construction of ovariectomized (OVX) and estradiol-implanted sheep model and light control experiment were previously described in detail (2, 12). Ewes were ovariectomized and estradiol-implanted (E2, Sigma Chemical Co., St. Louis, MO, USA) to maintain plasma estradiol levels of 3–5 pg/ml (6, 16) in October, 2016, according to the model developed by Karsch et al. (1984) (17). This OVX + E2 model normalizes the level of circulating E2 (12), which uncovers the well-documented central seasonal shift in the negative feedback action of E2 on gonadotropin secretion (18). After the surgery, the ewes recovered for 30 days before artificial light control. Then, in November 2016, all ewes were brought indoors and submitted to a light program simulating the outdoor photoperiodic condition by time-control switch. Firstly, all ewes were kept in artificial SP with lights on during 10:30–18:30 (SP, 8:16 h light/dark) for 21 days and switched to LP with lights on during 06:30–22:30 (LP, 16:8 h light/dark) for 42 days, with free access to water and food. Ewes were euthanized [intravenous pentobarbital (100 mg/kg)] at ZT4 [4 h after lights on] of SP21, LP7, LP21, and LP42 (8, 19–21). Consistent with the specific location described by Wood et al. (7) and Lomet et al. (6), the PT tissue (Figure 1) of each ewe was immediately collected and stored at −80°C for total RNA extraction.

The authors apologize for all above errors and state that this does not change the scientific conclusions of the article in any way. The original article has been updated.

## Publisher's Note

All claims expressed in this article are solely those of the authors and do not necessarily represent those of their affiliated organizations, or those of the publisher, the editors and the reviewers. Any product that may be evaluated in this article, or claim that may be made by its manufacturer, is not guaranteed or endorsed by the publisher.

